# Expression-Based *In Silico* Screening of Candidate Therapeutic Compounds for Lung Adenocarcinoma

**DOI:** 10.1371/journal.pone.0014573

**Published:** 2011-01-21

**Authors:** Guiping Wang, Yun Ye, Xiaoqin Yang, Hongying Liao, Canguo Zhao, Shuang Liang

**Affiliations:** 1 Bioinformatics Group, Institute of Genetic Engineering, Southern Medical University, Guangzhou, People's Republic of China; 2 Guangzhou Medical College, Guangzhou, People's Republic of China; 3 Department of Biological and Chemical Engineering, Guangxi University of Technology, Liuzhou, People's Republic of China; 4 Third Affiliated Hospital of Sun Yat-Sen University, Guangzhou, People's Republic of China; Deutsches Krebsforschungszentrum, Germany

## Abstract

**Background:**

Lung adenocarcinom (AC) is the most common form of lung cancer. Currently, the number of medical options to deal with lung cancer is very limited. In this study, we aimed to investigate potential therapeutic compounds for lung adenocarcinoma based on integrative analysis.

**Methodology/Principal Findings:**

The candidate therapeutic compounds were identified in a two-step process. First, a meta-analysis of two published microarray data was conducted to obtain a list of 343 differentially expressed genes specific to lung AC. In the next step, expression profiles of these genes were used to query the Connectivity-Map (C-MAP) database to identify a list of compounds whose treatment reverse expression direction in various cancer cells. Several compounds in the categories of HSP90 inhibitor, HDAC inhibitor, PPAR agonist, PI3K inhibitor, passed our screening to be the leading candidates. On top of the list, three HSP90 inhibitors, i.e. 17-AAG (also known as tanespimycin), monorden, and alvespimycin, showed significant negative enrichment scores. Cytotoxicity as well as effects on cell cycle regulation and apoptosis were evaluated experimentally in lung adenocarcinoma cell line (A549 or GLC-82) with or without treatment with 17-AAG. In *vitro* study demonstrated that 17-AAG alone or in combination with cisplatin (DDP) can significantly inhibit lung adenocarcinoma cell growth by inducing cell cycle arrest and apoptosis.

**Conclusions/Significance:**

We have used an *in silico* screening to identify compounds for treating lung cancer. One such compound 17-AAG demonstrated its anti-lung AC activity by inhibiting cell growth and promoting apoptosis and cell cycle arrest.

## Introduction

Lung cancer, including small cell lung cancer (SCLC) and non-small cell lung cancer (NSCLC), is the leading cause of cancer deaths for both men and women worldwide, particularly in China [1],[2],[3],[4]. Lung adenocarcinoma is the predominant histological subtype of NSCLC and accounts for about 20∼30% of primary lung cancer cases for people under the age of 45 regardless of smoking history [5]. Clinically, surgical resection remains the most effective treatment for early-stage NSCLC patients (stage I–II), with 30%–60% of survival 5 years after intervention [6]. However, five-year survival rate drops to about 10∼15% for most NSCLC patients due to late diagnosis, when the tumor has become unresectable. Chemotherapy using cisplatin (DDP) in combination with other antitumor agents (e.g., paclitaxel, gemcitabine, vinorelbine, etc.) remains the first treatment plan for advanced NSCLC. In recent years, the use of some small molecular agents targeting specific tyrosine kinases of cancer cells shows favorable results, but the improvement is often insignificant to extend the lives of NSCLC patients. Thus, there is a need for finding new and effective therapeutic agents for lung adenocarcinoma.

Gene expression profiling is used as a powerful tool for elucidating disease-specific molecular mechanism, biological pathway [7], as well as for predicting drug response or resistance [8], disease outcome [9], and for discovering new targets [10]. Recently, Lamb and his coworkers [11] created a searchable database (“Connectivity Map”, C-MAP) containing thousands of gene-expression signatures of various cultured cancer cells exposed to a large collection of small molecule compounds. C-MAP represents a useful tool for the discovery of unexplored connections among small molecules, diseases, and the biological pathways that join them. By comparing expression signatures, C-MAP serves as a proxy to search for new indications of all compounds surveyed, and has seen its success in drug re-discovery. Using C-MAP, Guo *et al* identified rapamycin as a potential glucocorticoid resistance reversal agent [12]. Two new hsp90 inhibitors, celastrol and gedunin, were discovered using this approach [13]. In another study, new therapeutic compounds for treating neuroblastoma were similarly identified [14]. More researches have demonstrated its potential [15],[16].

In the present study, we set out to discover agents not known for targeting lung adenocarcinoma by an expression-based *in silico* screening. We screened and ranked for genes differentially expressed in lung adenocarcinoma versus normal lung tissue. The ranked gene list (denoted as signature) was then submitted to the C-Map database for the identification of compounds or drugs reversing the expression direction of the signature. Among the candidate compounds found, 17-AAG (also known as Tanespimycin) was selected as a potential therapeutic agent for lung adenomcarcinoma. In subsequent validation experiments, 17-AAG alone or in combination with cisplatin inhibited lung adenocarcinoma cell proliferation and induced both cell cycle arrest and apoptosis.

## Results

### Genes differentially expressed between lung adenocarcinoma and normal lung tissue

C-MAP can be used to query gene expression signature against a collection of microarray expression signatures from cultured disease-borne human cell lines treated with bioactive small molecule compounds. Here, we tested whether C-MAP could be used to identify compounds reversing the expression signature of lung adenocarcinoma.

The workflow of the meta-analysis of multiple microarray data sets is shown in Supplementary Figure. S1. In brief, we first defined a gene expression signature of lung adenocarcinoma by identifying differentially-expressed genes common to the two data sets used. 343 such differentially expressed genes with at least a 2-fold change found by the meta-analysis were used to define a lung AC signature (Supplementary Figure. S2). This signature includes 93 up-regulated and 250 down-regulated genes. A detailed gene list can be found in Supplementary Table S2. Gene Set Enrichment Analysis (GSEA) suggested that several pathways related to CELL_CYCLE, AKT, PPARA and TIGHT_JUNCTION regulation were dysfunctional in lung AC (unpublished result).

### Identification of compounds reverting expression signature of lung adenocarcinoma

Using a simple pattern-matching algorithm, C-MAP links drugs, genes and diseases by measuring similarity or dissimilarity in gene-expression. To identify drugs exerting antitumor effects by causing a reversal of the gene expression signature of lung adenocarcinoma to a favorable one, we performed C-MAP analysis by searching for negatively-correlated gene expression patterns associated with drug-treated cancer cells [11] . The expression signature of lung adenocarcinoma described above was used as input query to compare with those produced from drug treatments in the C-MAP database. Multiple drugs were identified for having expression signatures inverse-correlated with that of lung adenocarcinoma beyond chance. The results were summarized in Table 1. On top of the list, three HSP90 inhibitors, i.e. 17-AAG, monorden, and alvespimycin, showed significant negative enrichment.

**Table 1:** Results of Connectivity Map analysis.

Rank	Compound name	n	Enrichment	*p*	Drug category
1	vorinostat	7	−0.83	0	HDAC inhibitor
2	trichostatin A	92	−0.327	0	HDAC inhibitor
3	tanespimycin	36	−0.395	0.00006	**hsp90 inhibitor**
4	LY-294002	34	−0.31	0.00204	PI3K inhibitor
5	alvespimycin	7	−0.617	0.00443	**hsp90 inhibitor**
6	resveratrol	6	−0.655	0.00467	phytoalexin
7	thioridazine	4	−0.773	0.00539	antipsychotic
8	monorden	12	−0.469	0.00615	**hsp90 inhibitor**
9	15-delta prostaglandin J2	8	−0.552	0.00784	PPAR agonist
10	troglitazone	4	−0.675	0.02487	PPAR agonist
11	CP-690334-01	4	−0.673	0.02558	Not assessed
12	geldanamycin	10	−0.43	0.03386	**hsp90 inhibitor**
13	carbamazepine	5	−0.585	0.03609	anticonvulsant
14	pioglitazone	5	−0.571	0.04348	PPAR agonist
15	0173570-0000	4	−0.632	0.04492	Not assessed

NOTE: The compounds tested in at least four experiments were ranked based on *p* value.

### 17-AAG inhibited lung adenocarcinoma cell growth and enhanced cisplatin cytotoxicity *in vitro*

**Figure 1:**
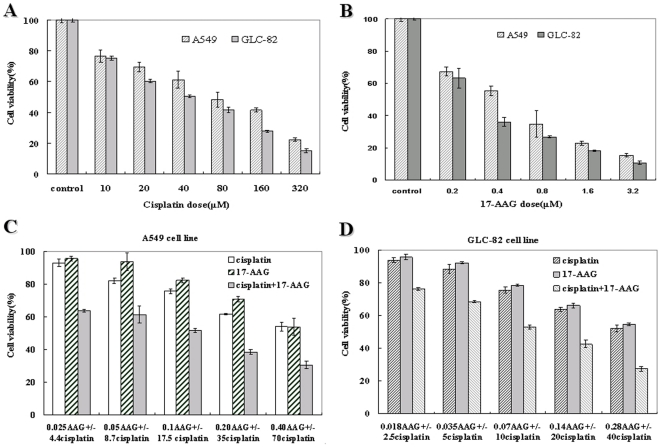
Cytotoxic effect of cisplatin (DDP), 17-AAG alone or together in lung adenocarcinoma cell lines. A549 or GLC-82 cells were incubated with 10∼320 µM cisplatin (**A**), 0.2∼3.2 µM 17-AAG (**B**), or various concentrations of cisplatin in combination with 17-AAG (**C, D**) at fixed ratio for 48 h. Cell viability was determined by the MTT assay and expressed as relative viability to control cells. Each bar represents results from triplicate experiments.

To investigate the biological effects of HSP90 inhibition, A549 or GLC-82 cells were cultured in medium containing various concentration of 17-AAG (0–3.2 µmol/L) or drug-free medium containing DMSO (0.1% final concentration) and cell viability was determined by the MTT assay. As shown in Figure 1A and 1B, it was evident that increasing concentrations of 17-AAG in the culture medium inhibited the growth of A549 or GLC-82 cells in a dose dependent manner. The IC_50_ of 17-AAG and cisplatin for A549 at 48 h was 0.454 and 69.63 µmol/L, for GLC-82 was 0.273 and 41.32 µmol/L, respectively. The combination of the two compounds was tested at fixed ratio based on their IC_50s_ for assessment of their synergy.

To evaluate the cytotoxic effects of combining 17-AAG and cisplatin in A549 or GLC-82 cells, we compared the growth inhibition resulted from single or combined treatment by the two compounds. As shown in Figure.1C and 1D, either 17-AAG or cisplatin alone inhibited the growth of A549 and GLC-82 cells in a concentration-dependent manner. The effect was greater when the two agents were combined, even at the lowest dosage combination. To determine whether the combination of cisplatin and 17-AAG in A549 or GLC-82 cells resulted in synergistic effects, the median effect method analysis of Chou and Talalay was used [17]. The combination index (CI) values are summarized in Table 2, all of which were below 1, indicating that there exists a synergistic antiproliferative effects between 17-AAG and cisplatin in A549 or GLC-82 cells.

**Table 2:** Synergy of 17-AAG with cisplatin in growth inhibition of A549 or GLC-82 cells.

A549 cell line			GLC-82 cell line		
17-AAG( µmol/L)	Cisplatin( µmol/L)	C I	17-AAG( µmol/L)	Cisplatin( µmol/L)	C I
0.025	4.4	0.321	0.018	2.5	0.423
0.05	8.7	0.339	0.035	5	0.482
0.1	17.5	0.420	0.07	10	0.597
0.2	35	0.570	0.14	20	0.684
0.4	70	0.697	0.28	40	0.855

NOTE: Combination Index (CI) values for 17-AAG with cisplatin at a constant ratio (1:175) as determined using the method of Chou and Talalay. CI = 1: additive effect; CI<1: synergy; CI>1: antagonism.

### 17-AAG caused cell cycle arrest and induced cell apoptosis in lung adenocarcinoma cells

**Figure 2:**
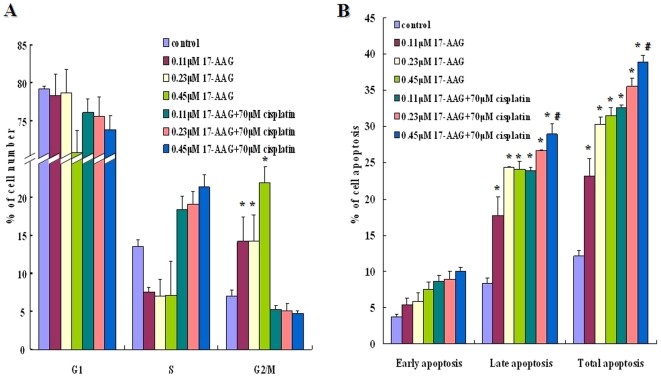
Effect of 17-AAG on cell cycle assessed using propidium iodide (PI) staining (A) and effect of 17-AAG on cell apoptosis quantitated using Annexin-V/PI detection (B). The A549 cells were exposed to various concentrations of 17-AAG or various concentrations of cisplatin in combination with 17-AAG at fixed ratio for 24 h. The cells were harvested and analyzed by flow cytometry. Each bar represents the means ± S.D (*n* = 3). Groups with significant change as compared to the respective control group were marked with asterisks (* *p*<0.05). Comparison of 17-AAG+cisplatin at their highest dosage was made against 17-AAG−treated groups and was found to be significant (# *p*<0.05) in terms of late/total apoptosis. G1: G1 phase; S: S phase; G2/M: G2/M phase.

HSP90 is known to be a chaperone for a variety of proteins that regulate cell cycle and apoptosis [18],[19] . Thus, we asked whether the antiproliferative activity of 17-AAG was due to cell cycle arrest, apoptosis, or both. As compared to untreated cells, A549 cells treated with 17-AAG showed a signifiantly increased arrest in G_2_/M phase (p<0.05) and a marginal decrease in S phase at 24 h (Figure 2 panel A). This suggested that 17-AAG induced cell cycle arrest by preventing A549 cells from entering mitosis. However, the combination of 17-AAG and cisplatin produce modest to marginal change in S or G2/M arrest as compared to the respective control groups (Figure 2 panel A).

Annexin-V/PI (propidium iodide) flow cytometric experiments were performed to determine if 17-AAG alone or in combination with cisplatin could induce A549 cell apoptosis. Viable cells with intact membranes exclude PI, whereas dead and damaged cells with broken membranes are permeable to PI. As shown in Figure.2 panel B, upto 32% of cells treated with 17-AAG became apoptotic (including early and late apoptosis) as compared to about 12% apoptotic cells in control (*p*<0.05) (Figure.2 panel B). When 17-AAG combined with cisplatin, the percentage of late apoptotic cells, notably total apoptotic cells, increased as compared to those treated with 17-AAG alone (*p*<0.05) (Figure 2 panel B).

### Effects of 17-AAG on the expression of *EGFR*, *HIF 1A*, *AKT1* and *RAF-1* mRNA

**Figure 3:**
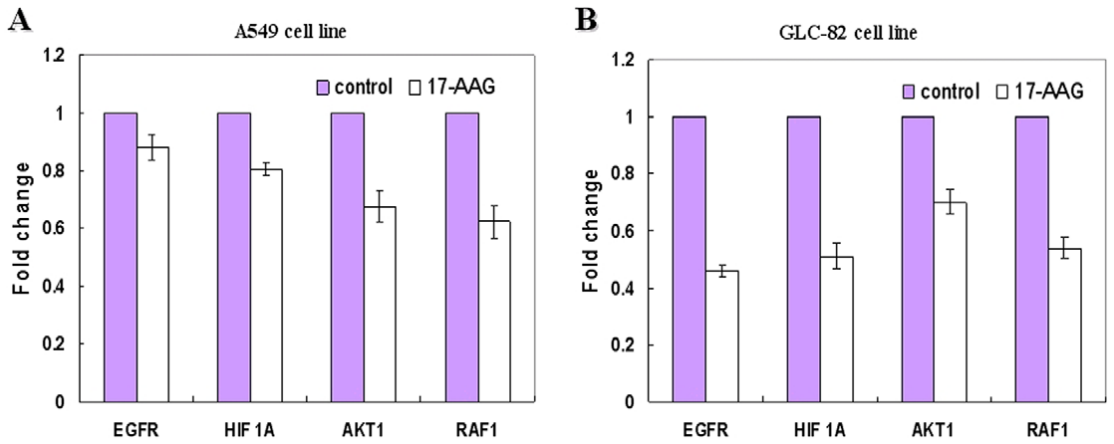
Expression of *EGFR, HIF1A, AKT1* and *RAF1* mRNA was determined by real time RT-PCR after A549 (A) or GLC-82 (B) cells were treated with 17-AAG or DMSO for 24 h.

Many factors including EGFR, HIF-1A, AKT1 and RAF-1 are known to be regulated by Hsp90 and their abnormal expression level is often associated with lung cancers [20],[21],[22],[23],[24]. We assessed the transcription levels of *EGFR, HIF-1A, AKT1* and *RAF-1* by real-time RT-PCR after A549 or GLC-82 cells were treated with 17-AAG or DMSO for 24 h. Results showed that the mRNA levels of *EGFR, HIF-1A, AKT1* and *RAF-1* in 17-AAG-treated A549 or GLC-82 cells decreased over control (Figure 3A and 3B). 17-AAG down-regulated expression of *EGFR* and *HIF1A* in GLC-82 cells by as much as 1.81 and 1.54-fold respectively as compared to those in A549 cells. However, the levels of *Raf1* and *AKT1* mRNA down-regulated by 17-AAG was similar in both cell lines.

## Discussion

Using an expression signature specific to lung adenocarcinoma, a number of compounds from C-MAP analysis were identified for having negatively-correlated effects on expression of query signature. These include HSP90 inhibitors, HDAC inhibitors, PPAR agonists, PI3K inhibitors, etc (Table 1). Some of the top hits in our initial screening, including histone deacetylase inhibitor trichostatin A [25], peroxisome proliferator-activated receptor agonist 15-delta prostaglandin J2, and PI3K inhibitor LY-294002, all have been shown to possess promising therapeutic activity for treating many cancer types inluding lung cancer [26],[27],[28],[29]. 17-AAG, one of the three top-ranked HSP90 inhibitors (17-AAG, monorden and alvespimycin), prevented proliferation of lung AC, induced G_2_/M cell cycle arrest and apoptosis in subsequent validation experiments as expected. When combined with the commonly-prescribed cisplatin, 17-AAG also showed synergistic interaction in inhibiting cell proliferation. These results agree with the rational behind our approach in finding new uses of existing compounds for unexplored medical conditions. In fact, this approach has been proved to be valuable in the area of drug discovery by others [12],[13],[14].

The constitutively action of PI3K/Akt signal transduction pathway has been reported to promote survival and proliferation of NSCLCs [23],[30],[31]. *Akt*, a downstream target of PI3k, is often mutated and amplified in a variety of human tumors including about 50% of NSCLC tissues [30]. *C-RAF (Raf-1)*, which is a component of the RAS/RAF/MEK/ERK pathway, also overexpressed in NSCLCs [32]. The alterations of some transmembrane receptors or signaling factors may result in the activation of PI3K/Akt signal pathway. For example, EGFR, which overexpressed in 40–80% of NSCLC, is an important up-stream regulator of PI3K/Akt [23] and RAS/RAF/MEK/ERK pathway in lung cancers [32]. In addition, the stabilization and activation of hypoxia-inducible transcription factor-1 (HIF-1), which contributed to the promotion of angiogenesis and the therapeutic resistance of tumor cells, can be affected by RAS/RAF/MEK/ERK and PI3K/Akt signal transduction pathways [21].

Hsp90 is a highly conserved molecular chaperone important for regulating a subset of cellular proteins. For example, it is critical for the maturation and conformational stabilization of proteins of normal cellular functions and those implicated in oncogenesis (e.g., *Akt*, *HER2*, *Raf-1, HIF-1A, EGFR* and *Cdk4*) [22],[33]. We speculate that 17-AAG exercises its inhibitory effect by reducing Hsp90 proteins activity and thereby destabilizing proteins important for cancer cell growth. Correlated with the observed growth inhibition, 17-AAG caused down-regulation of *EGFR, HIF-1A, AKT1* and *RAF1*, with a much deeper inhibition of *EGFR* and *HIF-1A* expression in GLC-82 than that in A549. Previous studies have demonstrated that various Hsp90 inhibitors (e.g.17-AAG, 17DMAG) caused the inhibition and interference of oncogenic signaling cascades in other advanced cancers by degrading EGFR, Akt, Raf-1 and HIF-1A, or by decreasing their expression [21],[23],[30],[32]. Here, we demonstrated that 17-AAG has similar effect in lung AC cells (Fig. 3A and B), which may result in growth inhibition, cell cycle arrest and apoptosis.

As shown in this study, A549 cells were found to arrest in G_2_/M after exposure to 17-AAG. The overall effect of 17-AAG on cell cycle regulation depends on cancer type or even cell lines, a reminiscence of G_1_ or G_2_/M arrest or both seen in different types of cancer cell lines [34]. In prostate cancer cell line, 17-AAG induced G_1_ arrest by degradating HER2, Akt, and androgen receptor [24]. In two different hepatoma cell lines, 17-AAG induced G_1_ and G_2_/M arrest in HuH7 and arrest only in G_2_/M in Hep3B cell lines, which owed to the difference of Akt expression in these cells [35]. However, 17-AAG and cisplatin have no synergy on cell cycle inhibition, which might be resulted from 17-AAG's effect being masked by cisplatin's effect in the preceding S phase.

Identifying new compounds for medical conditions is generally time-consuming and very expensive. We explore an *in silico* strategy to discover new uses of existing compounds for unmet clinical needs. A pre-requisite for the success of this approach is the availability of a high quality expression signature. This signature should mirror the changes between normal and diseased states to a reasonably good degree. To reduce the risk of bias, we selected our signature through meta-analysis. Meta-analysis provides more analytical power for us to generate such a more representative signature. Another major hurdle is the coverage of C-Map which currently contains over 7000 expression signatures with about 1300 compounds tested for four cell types. This may not be enough to deal with the complexity of many human diseases. In addition, only limited number of genes are allowed as input. This may distort pattern matching process if bias is present. When evaluating screening result, one needs to bear in mind that the connectivity score is merely a statistical measure of similarity or dissimilarity, as it is easier to obtain higher connectivity scores in a relatively low number of experimental instances. To be on the safe side, we initially filtered the compounds tested less than four times, and prioritized candidate compounds based on both *p-*value and the number of compounds in each class (Table 1).

In summary, our study demonstrated that gene expression signature-based *in silico* drug discovery is potentially valuable for the identification of new indications of existing compounds, which is critical for translational research and clinical applications. One major advantage of such approach is that the time-to-market is much shorter and cost-saving is significant as compared to new drug development since many compounds assayed in C-Map are approved by the Food and Drug Administration. Any promising drug(s) from such screen could be particularly beneficial to patients whose medical conditions have no effective treatment. 17-AAG is currently being evaluated for the treatment of multiple cancer indications in Phase I and Phase II clinical trials. Its anti-tumor activity in lung cancer has not been included in on-going trials but could be verified in subsequent trials, subjecting to more in-depth studies and structural optimization.

## Materials and Methods

### Compounds and Cell culture

17-AAG (17-Allylamino-17-demethoxygeldanamycin), obtained from Sigma- Aldrich (St. Louis, MO), was dissolved in dimethylsulfoxide (DMSO) to a 10 mMol/L stock concentration and stored at −20°C. The maximum volume (%) of DMSO in the experiment was less than 0.1%, and equal concentrations of DMSO alone served as a control in all experiments. Water-soluble cisplatin (DDP), also from Sigma-Aldrich (St. Louis, MO), was dissolved in PBS to a concentration of 0.1 mol/L and stored at −20°C. Two human lung adenocarcinoma cell lines A549 and GLC-82 (see Table S3 for more details) were obtained from GuangZhou Medical College cell repository and SUN YAT-SEN University cell repository, respectively. Cells were cultured in RPMI1640 medium supplemented with 10% fetal bovine serum (Invitrogen-Life Technologies, Inc.) at 37°C in the presence of 5% CO_2_.

### Acquisition and analysis of public microarray data

Raw data (.Cel files) of two published microarray data (GSE7670 and GSE10072) used in this study were obtained from the National Center for Biotechnology Information (NCBI) Gene Expression Omnibus (GEO) web site (http://www.ncbi.nlm.nih.gov/geo).

Details of the two microarray datasets are summarized in Supplementary Table S1. Microarray analysis was done with the BRB Array Tools (Version: 3.7.0), developed by the Biometric Research Branch of the US National Cancer Institute (http://linus.nci.nih.gov/BRB-ArrayTools.html). Two-sample T-test was used to identify differential genes. To control type I error, a total of 2,000 permutations were performed to set an upper limit of false discovery rate (FDR) to <1% at 95% confidence level. Differential expression was considered significant using a 2-fold change cutoff. Finally, differential probe IDs common to the two data sets were obtained as the lung AC signature for further C-MAP analysis.

### Connectivity Map analysis

C-Map (build02, http://www.broad.mit.edu/cmap/) contains more than 7,000 expression signatures representing 1,309 compounds. Up and down-regulated gene groups were submitted simultaneously to C-MAP for analysis. Enrichment scores for each and every compound in the database were computed using the gene set enrichment analysis algorithm [11]. Compounds with negative connectivity scores, which imply a mode of action by the matched compounds to reverse the expression direction of query genes in lung adenocarcinoma, were recorded as potential therapeutic agents for lung adenocarcinoma.

### Cell viability and toxicity assay

To evaluate cytotoxic effects of 17-AAG on lung adenocarcinoma cells, the 3-(4,5-dimethylthiazol-2-yl)-2,5-diphenyltetrazo-lium bromide assay (MTT) was performed as previously described [12]. In brief, A549 cells or GLC-82 (4×10^4^ cells/ml) were seeded in triplicate into 96-well plates (Costar, Corning, NY). After overnight incubation, cells were incubated in drug-free medium, or medium containing various concentrations of 17-AAG, or 17-AAG in combination with cisplatin for 48 h at 37°C. After drug exposure for the indicated concentrations and times, cells were incubated at 37°C for 4 h with the addition of 10 µl of MTT labeling reagent. Following MTT incubation, the absorbance of the samples was determined by a microplate reader at 490 nm (Tecan Sunrise, Switzerland). All experiments were performed at least three times for each experimental condition, and results were shown as relative ratios of viability in the treated over control groups. To confirm the synergistic cytotoxic interaction effect of cisplatin and 17-AAG, the combination index (CI) was calculated by Calcusyn Software (Biososoft, Ferguson, MO) according to the Chou-Talala method [12],[13]. Combination index values less than 1, equal to 1, or more than 1 indicate synergistic, additive, or antagonistic cytotoxic drug interactions, respectively.

### Cell cycle and cell apoptosis assays

Cell cycle and apoptosis assays were done as previously described [35]. In brief, cells were plated in duplicate into 6-well microplates at 5×10^6^ cells/well, and incubated in drug-free medium or medium containing 17-AAG, or 17-AAG plus cisplatin of varying concentrations at 37°C for 24 h. For cellular DNA content assay, cells were collected and washed with ice-cold PBS, fixed with 70% ethanol at 4°C for 1 h. After washing, cells were treated with RNase (0.25 mg/mL) for 30 min and stained with 50 g/mL of propidium iodide (PI). Stained cells were kept on ice and protected from light. Cell cycle analysis was performed with FACScan flow cytometer (Becton Dickinson, USA) and the percentage of cells in the G_1_, S and G_2_/M phases of the cell cycle was determined using the ModfitLT software program (Becton Dickinson). For Annexin V staining, cells were washed once with PBS and then 10 µL of Annexin V-FITC solution and 5 µL of PI solution were added. After 15 minutes of incubation away from light, cells were directly analyzed by FACScan and evaluated by the CellQuest program.

### Real-Time reverse transcription-PCR

A549 or GLC-82 cells were treated with 0.45 µM 17-AAG or DMSO for 24 h and total RNAs were isolated with Trizol (Invitrogen, Karlsruhe, Germany), 18S rRNA gene was used as the internal normalization control. Real-time RT-PCR(qRT-PCR) was performed on an ABI Prism 7300 Sequence Detection System (Applied Biosystems) in a 96-well reaction plate according to the manufacturer's recommendations. The PCR amplification protocol was as follows: 95°C for 5 min, followed by 40 cycles of 95°C for 15 s, 60°C for 15 s and 72°C for 30 s. Each PCR reaction was performed in triplicate and the experiments were repeated three times. PCR product quality was monitored using post-PCR melt curve analysis. Fold inductions were calculated using the formula 2-^(**△△**ct)^, where △△Ct is △Ct_(treatment)_ −△Ct_(control)_, △Ct is Ct_(target gene)_ −Ct_(18sRNA)_ and Ct is the cycle at which the threshold is crossed [36]. The primers used for real-time PCR are as follows:

*EGFR*: 5-ACTACAGGTCAAGTGGTAGC-3(forward) and

5- GAGGAGGAGTATGTGTGAAGGA -3 (reverse)

*HIF 1A:* 5- GTGGATTACCACAGCTGA -3 (forward) and

5- GCTCAGTTAACTTGATCCA -3(reverse)

*AKT1:* 5- TCTGTCACCAGCTATCTG -3(forward) and

5-GACAGTCACCAAGAACTG-3(reverse);

*RAF-1:* 5- CTGCTTTGGTACTATGGAAC-3 (forward) and

5- TTCAGCATGATGGAAGACTG -3 (reverse)

18srRNA: 5- CCTGGATACCGCAGCTAGGA-3 (forward) and

5- GCGGCGCAATACGAATGCCCC -3 (reverse)

### Statistical analysis

The statistical significance of cell cycle distributions and apoptosis between groups were assessed with one-way ANOVA followed by post-hoc LSD and Dunnett T3 test using the SPSS software (version 13.0). *p* values of <0.05 were considered to be significant.

## Supporting Information

Figure S1 The workflow of the meta-analysis of microarray data sets. Meta-analysis was done with the BRB Array Tools. Intensity filtering was used in individual arrays for quality-control purposes before arrays are normalized. Signal intensity threshold was set to 1 by default. The median-normalization was used for data normalization. Class comparison between groups of arrays was used to find genes that are differentially expressed between two phenotype classes, whereas two-sample T-test was used as type of univariate test.
(1.61 MB TIF)

Figure S2 Venn diagram for the resultant genes. Analysis of GSE7670 produced 434 differential genes (125 up and 309 down), and analysis of GSE10072 identified 530 differential genes (180 up and 350 down). 343 genes (A) were found common from the two result sets, including 93 up-regulated (B) and 250 down-regulated genes (C).
(1.51 MB TIF)

Table S1 Summary of the two microarray datasets, GSE7670 and GSE10072. There are 66 samples in dataset GSE7670, 107 samples in dataset GSE10072. We selected 54 paired samples from 27 patients in dataset GSE7670, 62 paired samples from 31 patients in dataset GSE10072.
(0.03 MB XLS)

Table S2 Lung adenocarcinoma signature genes. This signature includes 93 up-regulated and 250 down-regulated genes.
(0.05 MB XLS)

Table S3 Cytogenetic information of two human lung adenocarcinoma cell lines, GLC-82 and A549.
(0.03 MB DOC)

## References

[1] Yang L, Parkin DM, Ferlay J, Li L, Chen Y (2005). Estimates of cancer incidence in China for 2000 and projections for 2005.. Cancer Epidemiol Biomarkers Prev.

[2] Yang L, Parkin DM, Li L, Chen Y (2003). Sources of information on the burden of cancer in China.. Asian Pac J Cancer Prev.

[3] Yang L, Parkin DM, Li LD, Chen YD, Bray F (2004). Estimation and projection of the national profile of cancer mortality in China: 1991–2005.. Br J Cancer.

[4] Yang L, Parkin DM, Whelan S, Zhang S, Chen Y (2005). Statistics on cancer in China: cancer registration in 2002.. Eur J Cancer Prev.

[5] Crocetti E, Paci E (2002). Trends in lung adenocarcinoma incidence and survival.. Lung Cancer.

[6] Mountain CF (1997). Revisions in the International System for Staging Lung Cancer.. Chest.

[7] Riedel RF, Porrello A, Pontzer E, Chenette EJ, Hsu DS (2008). A genomic approach to identify molecular pathways associated with chemotherapy resistance.. Mol Cancer Ther.

[8] Potti A, Dressman HK, Bild A, Riedel RF, Chan G (2006). Genomic signatures to guide the use of chemotherapeutics.. Nat Med.

[9] Pomeroy SL, Tamayo P, Gaasenbeek M, Sturla LM, Angelo M (2002). Prediction of central nervous system embryonal tumour outcome based on gene expression.. Nature.

[10] Rahman KW, Li Y, Wang Z, Sarkar SH, Sarkar FH (2006). Gene expression profiling revealed survivin as a target of 3,3′-diindolylmethane-induced cell growth inhibition and apoptosis in breast cancer cells.. Cancer Res.

[11] Lamb J, Crawford ED, Peck D, Modell JW, Blat IC (2006). The Connectivity Map: using gene-expression signatures to connect small molecules, genes, and disease.. Science.

[12] Wei G, Twomey D, Lamb J, Schlis K, Agarwal J (2006). Gene expression-based chemical genomics identifies rapamycin as a modulator of MCL1 and glucocorticoid resistance.. Cancer Cell.

[13] Hieronymus H, Lamb J, Ross KN, Peng XP, Clement C (2006). Gene expression signature-based chemical genomic prediction identifies a novel class of HSP90 pathway modulators.. Cancer Cell.

[14] De Preter K, De Brouwer S, Van Maerken T, Pattyn F, Schramm A (2009). Meta-mining of neuroblastoma and neuroblast gene expression profiles reveals candidate therapeutic compounds.. Clin Cancer Res.

[15] Kutalik Z, Beckmann JS, Bergmann S (2008). A modular approach for integrative analysis of large-scale gene-expression and drug-response data.. Nat Biotechnol.

[16] Li Y, Hao P, Zheng S, Tu K, Fan H (2008). Gene expression module-based chemical function similarity search.. Nucleic Acids Res.

[17] Chou TC, Talalay P (1984). Quantitative analysis of dose-effect relationships: the combined effects of multiple drugs or enzyme inhibitors.. Adv Enzyme Regul.

[18] Takayama S, Reed JC, Homma S (2003). Heat-shock proteins as regulators of apoptosis.. Oncogene.

[19] Young JC, Moarefi I, Hartl FU (2001). Hsp90: a specialized but essential protein-folding tool.. J Cell Biol.

[20] Georgakis GV, Li Y, Younes A (2006). The heat shock protein 90 inhibitor 17-AAG induces cell cycle arrest and apoptosis in mantle cell lymphoma cell lines by depleting cyclin D1, Akt, Bid and activating caspase 9.. Br J Haematol.

[21] Kim WY, Oh SH, Woo JK, Hong WK, Lee HY (2009). Targeting heat shock protein 90 overrides the resistance of lung cancer cells by blocking radiation-induced stabilization of hypoxia-inducible factor-1alpha.. Cancer Res.

[22] Neckers L, Ivy SP (2003). Heat shock protein 90.. Curr Opin Oncol.

[23] Sawai A, Chandarlapaty S, Greulich H, Gonen M, Ye Q (2008). Inhibition of Hsp90 down-regulates mutant epidermal growth factor receptor (EGFR) expression and sensitizes EGFR mutant tumors to paclitaxel.. Cancer Res.

[24] Solit DB, Zheng FF, Drobnjak M, Munster PN, Higgins B (2002). 17-Allylamino-17-demethoxygeldanamycin induces the degradation of androgen receptor and HER-2/neu and inhibits the growth of prostate cancer xenografts.. Clin Cancer Res.

[25] Mukhopadhyay NK, Weisberg E, Gilchrist D, Bueno R, Sugarbaker DJ (2006). Effectiveness of trichostatin A as a potential candidate for anticancer therapy in non-small-cell lung cancer.. Ann Thorac Surg.

[26] Ebi H, Tomida S, Takeuchi T, Arima C, Sato T (2009). Relationship of deregulated signaling converging onto mTOR with prognosis and classification of lung adenocarcinoma shown by two independent in silico analyses.. Cancer Res.

[27] Han H, Shin SW, Seo CY, Kwon HC, Han JY (2007). 15-Deoxy-delta 12,14-prostaglandin J2 (15d-PGJ 2) sensitizes human leukemic HL-60 cells to tumor necrosis factor-related apoptosis-inducing ligand (TRAIL)-induced apoptosis through Akt downregulation.. Apoptosis.

[28] Pitt SC, Chen H, Kunnimalaiyaan M (2009). Phosphatidylinositol 3-kinase-Akt signaling in pulmonary carcinoid cells.. J Am Coll Surg.

[29] Shin SW, Seo CY, Han H, Han JY, Jeong JS (2009). 15d-PGJ2 induces apoptosis by reactive oxygen species-mediated inactivation of Akt in leukemia and colorectal cancer cells and shows in vivo antitumor activity.. Clin Cancer Res.

[30] Solit DB, Basso AD, Olshen AB, Scher HI, Rosen N (2003). Inhibition of heat shock protein 90 function down-regulates Akt kinase and sensitizes tumors to Taxol.. Cancer Res.

[31] Crowell JA, Steele VE (2003). AKT and the Phosphatidylinositol 3-Kinase/AKT Pathway: Important Molecular Targets for Lung Cancer Prevention and Treatment.. Journal of the National Cancer Institute.

[32] da Rocha Dias S, Friedlos F, Light Y, Springer C, Workman P (2005). Activated B-RAF is an Hsp90 client protein that is targeted by the anticancer drug 17-allylamino-17-demethoxygeldanamycin.. Cancer Res.

[33] Senju M, Sueoka N, Sato A, Iwanaga K, Sakao Y (2006). Hsp90 inhibitors cause G2/M arrest associated with the reduction of Cdc25C and Cdc2 in lung cancer cell lines.. J Cancer Res Clin Oncol.

[34] Okamoto J, Mikami I, Tominaga Y, Kuchenbecker KM, Lin YC (2008). Inhibition of Hsp90 leads to cell cycle arrest and apoptosis in human malignant pleural mesothelioma.. J Thorac Oncol.

[35] Watanabe G, Behrns KE, Kim JS, Kim RD (2009). Heat shock protein 90 inhibition abrogates hepatocellular cancer growth through cdc2-mediated G2/M cell cycle arrest and apoptosis.. Cancer Chemother Pharmacol.

[36] Whyte L, Huang YY, Torres K, Mehta RG (2007). Molecular mechanisms of resveratrol action in lung cancer cells using dual protein and microarray analyses.. Cancer Res.

