# Supplementary figures and images for: Expression-Based In Silico Screening of Candidate Therapeutic Compounds for Lung Adenocarcinoma
Source: PLoS One. 2011 Jan 21;6(1):e14573. doi: 10.1371/journal.pone.0014573 (PMC3024967; doi:10.1371/journal.pone.0014573)

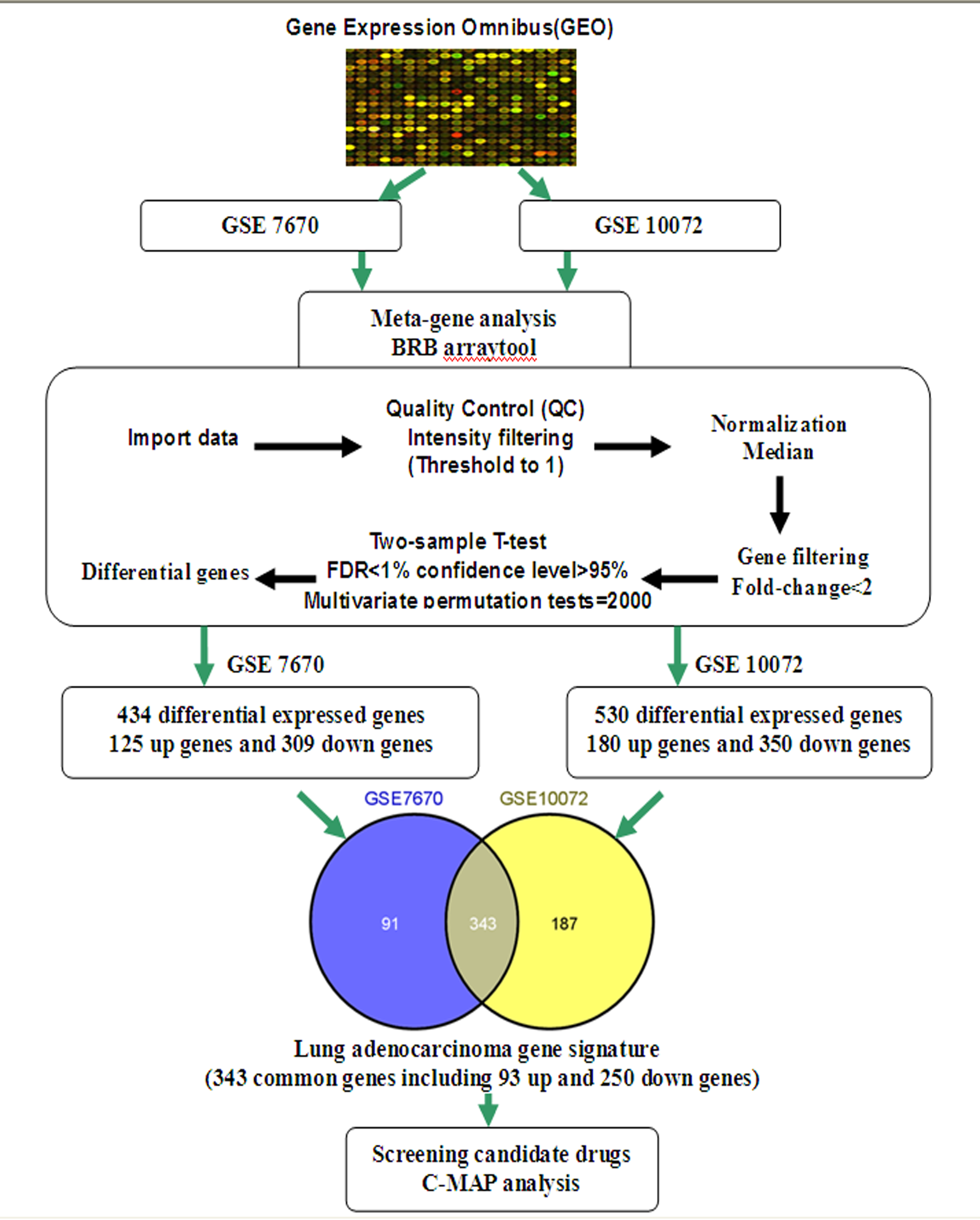

Supplement: Figure S1 — The workflow of the meta-analysis of microarray data sets. Meta-analysis was done with the BRB Array Tools. Intensity filtering was used in individual arrays for quality-control purposes before arrays are normalized. Signal intensity threshold was set to 1 by default. The median-normalization was used for data normalization. Class comparison between groups of arrays was used to find genes that are differentially expressed between two phenotype classes, whereas two-sample T-test was used as type of univariate test. (1.61 MB TIF) [file pone.0014573.s001.tif]

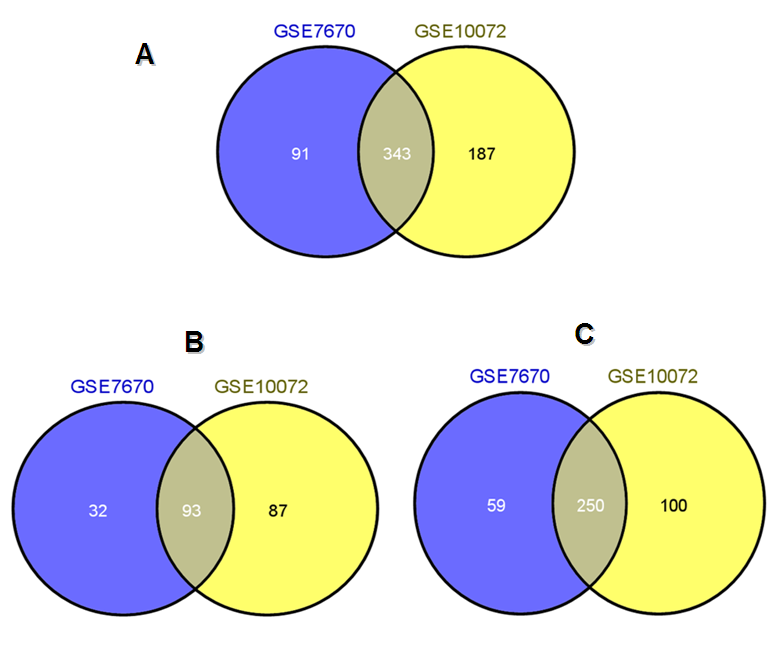

Supplement: Figure S2 — Venn diagram for the resultant genes. Analysis of GSE7670 produced 434 differential genes (125 up and 309 down), and analysis of GSE10072 identified 530 differential genes (180 up and 350 down). 343 genes (A) were found common from the two result sets, including 93 up-regulated (B) and 250 down-regulated genes (C). (1.51 MB TIF) [file pone.0014573.s002.tif]
