# Supplementary material for: Expression-Based In Silico Screening of Candidate Therapeutic Compounds for Lung Adenocarcinoma
Source: PLoS One. 2011 Jan 21;6(1):e14573. doi: 10.1371/journal.pone.0014573 (PMC3024967; doi:10.1371/journal.pone.0014573)
Supplement: Table S3 — Cytogenetic information of two human lung adenocarcinoma cell lines, GLC-82 and A549. (0.03 MB DOC) [file pone.0014573.s005.doc]

# Cytogenetic information of the two human lung adenocarcinoma cell lines, GLC-82 and A549 [1,2,3]

|  | GLC-82 cell line | A549 cell line |
| --- | --- | --- |
| Origin | GLC-82 cell line was established from the lung cancer tissue of a female retired worker who had suffered long-term exposure to environmental radon gas pollution. | A549 cell line was first developed in 1972 through the removal and culturing of cancerous lung tissue in the explanted tumor of 58-year-old Caucasian male |
| Chromosome numbers | 60-64 (40 normal and 24 abnormal) | 62-66(53 normal and 13 abnormal) |
| Character of  the normal chromosomes | Total: 40  seven monosomic (2, 5, 10, 14, 20, 21 and 22); twelve disomic (4, 6, 8, 9, 11, 12, 13, 15, 17, 18, 19 and X); three trisomic (1, 7 and 16). | Total: 53  three monosomic (1, 4, 12); fourteen disomic (3, 6, 9, 10, 11, 13, 15, 18-22, X and Y); six trisomic (2, 5, 7, 8, 14 and 16); one tetrasomic (17) . |
| Character of  the abnormal chromosomes | Total: 24  fourteen (G1-G4, G7, G12, G13, G15, G16, G18-G22) was derived from translocation; six (G5, G6, G11, G14, G17 and G24) was formed by deletion; four isochromosomes (one chromosome 20q and three copies of chromosome 5p). | Total: 13  six (A4, A6, A7, A9, A10 and A13) were formed by translocations; seven (A1-A3, A5, A8, A11 and A12) were derived by deletion or amplification. |
| *Tp53* status | Mutant *p53* | wild-type *p53* |

**Reference**

1. Fan YS, Li P (1987) Cytogenetic studies of four human lung adenocarcinoma cell lines. Cancer Genet Cytogenet 26: 317-325.

2. Liang MD (1985) [Establishment of lung adenocarcinoma cell line in Gejiu (GLC-82) and study of its biologic properties]. Zhonghua Zhong Liu Za Zhi 7: 81-82.

3. Peng KJ, Wang JH, Su WT, Wang XC, Yang FT, et al. (2010) Characterization of two human lung adenocarcinoma cell lines by reciprocal chromosome painting. Dongwuxue Yanjiu 31: 113-121.
